# A Novel Risk Score for Type 2 Diabetes Containing Sleep Duration: A 7-Year Prospective Cohort Study among Chinese Participants

**DOI:** 10.1155/2020/2969105

**Published:** 2020-01-04

**Authors:** Xiangtong Liu, Zhiwei Li, Jingbo Zhang, Shuo Chen, Lixin Tao, Yanxia Luo, Xiaolin Xu, Jason Peter Fine, Xia Li, Xiuhua Guo

**Affiliations:** ^1^School of Public Health, Capital Medical University, Beijing 100069, China; ^2^Beijing Municipal Key Laboratory of Clinical Epidemiology, Beijing, China; ^3^Beijing Physical Examination Center, Beijing 100077, China; ^4^The University of Queensland, Brisbane, Australia; ^5^University of North Carolina, Chapel Hill 462001, USA; ^6^La Trobe University, Melbourne, Australia

## Abstract

**Background:**

Sleep duration is associated with type 2 diabetes (T2D). However, few T2D risk scores include sleep duration. We aimed to develop T2D scores containing sleep duration and to estimate the additive value of sleep duration.

**Methods:**

We used data from 43,404 adults without T2D in the Beijing Health Management Cohort study. The participants were surveyed approximately every 2 years from 2007/2008 to 2014/2015. Sleep duration was calculated from the self-reported usual time of going to bed and waking up at baseline. Logistic regression was employed to construct the risk scores. Integrated discrimination improvement (IDI) and net reclassification improvement (NRI) were used to estimate the additional value of sleep duration.

**Results:**

After a median follow-up of 6.8 years, we recorded 2623 (6.04%) new cases of T2D. Shorter (both 6-8 h/night and <6 h/night) sleep durations were associated with an increased risk of T2D (odds ratio (OR) = 1.43, 95% confidence interval (CI) = 1.30-1.59; OR = 1.98, 95%CI = 1.63-2.41, respectively) compared with a sleep duration of >8 h/night in the adjusted model. Seven variables, including age, education, waist-hip ratio, body mass index, parental history of diabetes, fasting plasma glucose, and sleep duration, were selected to form the comprehensive score; the *C*-index was 0.74 (95% CI: 0.71-0.76) for the test set. The IDI and NRI values for sleep duration were 0.017 (95% CI: 0.012-0.022) and 0.619 (95% CI: 0.518-0.695), respectively, suggesting good improvement in the predictive ability of the comprehensive nomogram. The decision curves showed that women and individuals older than 50 had more net benefit.

**Conclusions:**

The performance of T2D risk scores developed in the study could be improved by containing the shorter estimated sleep duration, particularly in women and individuals older than 50.

## 1. Introduction

Type 2 diabetes (T2D) is arguably one of the largest epidemics globally [1]. The epidemic of T2D could threaten global development through premature morbidity and mortality from associated complications [2, 3]. With 702 million cases projected by 2045, identifying modifiable risk factors for T2D is of urgent public health importance [4].

More than half of T2D cases remain undiagnosed for many years before the onset of complications. It is critical to identify high-risk individuals, since early intervention can effectively prevent or delay them from developing T2D [5]. Traditional diagnostic tests (e.g., oral glucose tolerance test) are expensive and time-consuming and therefore not suitable for population screening. The most cost-effective method is to use T2D scores for risk stratification [6]. Researchers have built multivariate scores to help with risk assessment for decades.

Recently, sleep duration has emerged as a novel target for T2D prevention [7]. Sleep loss has become a major public health concern due to its high prevalence and association with mortality [8]. Previous studies on the association between sleep and T2D remain inconsistent. A recent Mendelian randomization study did not indicate a direct association between sleep duration and T2D [9]. However, other studies have shown that sleep duration is associated with T2D risk [10, 11]. In addition, few diabetes scores include sleep duration [12, 13], especially for T2D.

Furthermore, much less is known about the association between T2D risk and eating pace and taste preferences, although eating quickly may be an important predictor in T2D scores [14]. Eating speed is controllable, and eating slowly is an easy and acceptable target for lifestyle interventions to prevent T2D. Although limited, a large prospective cohort study showed that spicy preferences are associated with overall and specific mortality [15].

Thus, the aims of this article were (a) to develop T2D risk scores containing sleep duration to stratify T2D risk and identify high-risk populations and (b) to estimate the additive value of sleep duration taking eating pace and taste preferences into account.

## 2. Materials and Methods

### 2.1. Study Population

The Beijing Health Management Cohort (BHMC) study is an ongoing prospective cohort study that is aimed at investigating factors associated with metabolic disorders in healthy individuals from urban areas of north-eastern China. The BHMC study was based on cluster sampling from a working population of functional communities by performing health check-ups at the Beijing Physical Examination Center and Beijing Xiaotangshan Hospital. The participants participated in follow-up every two to three years with a general health questionnaire, a physical examination, and laboratory measurements. Details of the study design and recruitment methods have been described elsewhere [16].

**Figure 1:**
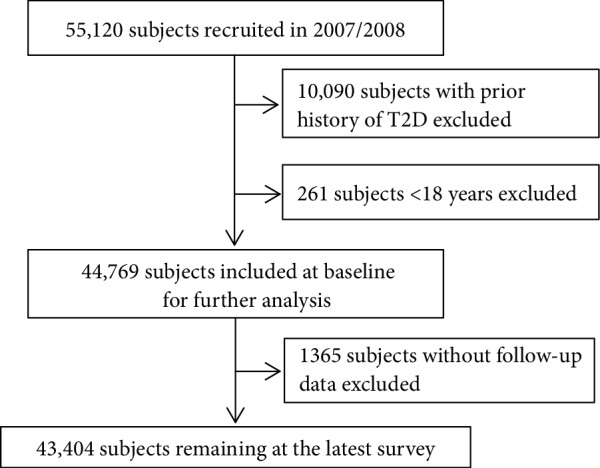
Study population selection flowchart.

In the present study, the 2007/2008 survey of the BHMC study was used as the starting point of the follow-up, and the 2014/2015 survey was used as the endpoint of the follow-up. A total of 55,120 individuals were recruited in this study in 2007/2008. Among these individuals, 10,090 subjects with a previous diagnosis of T2D and 261 subjects younger than 18 years were excluded from the study, leaving 44,769 subjects at baseline (81.22% response rate). According to recommendations from the American Diabetes Association, T2D at baseline was defined as a self-reported history of a T2D diagnosis, the use of antidiabetic medicine, or a measured fasting plasma glucose (FPG) level ≥ 7.0 mmol/L [6]. Approximately 3.05% of participants were lost to follow-up (*n* = 1365). The sensitivity analysis showed that there were no significant differences in the distributions of baseline characteristics between those lost to follow-up and those followed. Finally, 43,404 subjects (mean age: 36.79 ± 13.29 years; age range: 18–80 years; 55.31% males) were included in the present study (see Figure 1).

The study was approved by the Ethics Committee of the Capital Medical University of China, Beijing (reference no. 2013SY26). All participants signed an informed consent form, and all data used in the analyses were deidentified.

### 2.2. Outcomes

The main outcome was the incidence of T2D during the seven-year follow-up. According to recommendations from the American Diabetes Association, T2D was defined as a self-reported history of a T2D diagnosis, the use of antidiabetic medicine, or a measured FPG level of ≥7.0 mmol/L [6]. At each survey, participants were asked, “Have you ever been told by a doctor that you have T2D (high blood sugar)? If yes, how long ago were you diagnosed with T2D? Have you ever taken any antidiabetic medicine?” The participants' FPG levels were measured at each follow-up survey with the glucose hexokinase method.

The incidence of T2D was based on the first report. The date of diagnosis (incidence) was defined as the diagnosis date if a recent T2D history was reported by questionnaire or the examination date at which a new case of T2D was identified, whichever occurred first.

### 2.3. Covariates

There were four groups of covariates used in the analysis, and these covariates were collected at each survey. The covariates included were sociodemographic characteristics (age, sex, marital status, and education), lifestyle behaviours (sleep duration, smoking, drinking, physical activity, and work stress), dietary habit factors (eating pace, breakfast frequency, taste preferences, milk intake, grain intake, and dietary patterns), and other chronic conditions (obesity, hypertension, and hyperlipidaemia).

A general health questionnaire was used to obtain information about these covariates. Given that it is not feasible to accurately measure the actual sleep time using objective methods such as polysomnography in large studies, sleep duration was recorded according to participants' responses to the question “Over the past three months, what time have you normally gone to bed and woken up?” (with time recorded in a 24 h format). The estimated nocturnal sleep duration was defined as the time space between bedtime and waking time and was categorized into three levels: <6, 6 to 8, and >8 h per night. For the physical activity intensity variable, “high” was defined as swimming, playing ball, or skipping rope; “moderate” was defined as jogging, cycling, or climbing; and “mild” was defined as walking, tai chi, or dancing. According to the demand-control model, work stress was defined as high psychological demands (such as intense tasks) and low control (in areas such as skill discretion) at work [17]. In our study, work stress was categorized into four semiquantitative categories. The “small” group was defined as having neither high psychological demands nor low control at work, the “medium” group was defined as having one of them, the “high” group was defined as having both, and the “retired” group was the category specified for retired subjects.

In the present study, the speed of eating was self-reported by the response to the question “How fast is your speed of eating?” The responses were chosen from three semiquantitative categories: “slow,” “medium,” and “fast.” Previous reports have demonstrated good validity and adequate reproducibility of speed of eating, as assessed by self-report [18]. The measurement and classification of each variable category have been reported elsewhere in detail [16].

The resting heart rate was measured using a 12-lead electrocardiogram with the participants in the supine position [19]. Blood pressure was measured twice with a mercury sphygmomanometer and an appropriately sized cuff on the left arm of the seated participants; the average of the blood pressure measurements constituted the examination blood pressure value. The blood pressure measurements were at least 5 minutes apart. If the difference between the two measurements exceeded 5 mmHg, then the blood pressure was measured again, and the average of the three measurements was ultimately selected. Blood pressure was classified into two groups: high (systolic blood pressure > 140 mmHg or diastolic blood pressure > 90 mmHg) and normal.

Waist circumference, hip circumference, height, and weight were measured in the standing position without heavy clothing to the nearest 0.1 cm or 0.1 kg. BMI was calculated as kilograms divided by height in metre squared and categorized as normal weight (<24 kg/m^2^), overweight (24-27.9 kg/m^2^), or obese (≥28 kg/m^2^), according to the criteria for Asian populations [20]. The waist-hip ratio (WHR) was calculated as the waist circumference (cm) divided by the hip circumference (cm) and was further divided into four categories: <0.75 in men or <0.72 in women, 0.75 to 0.79 in men or 0.72 to 0.76 in women, 0.80 to 0.83 in men or 0.77 to 0.81 in women, and ≥0.84 in men or ≥0.82 in women.

Blood samples were collected from an antecubital vein in the morning after an overnight fasting period and placed in tubes containing EDTA. The samples were analysed immediately after pretreatment or stored at -80°C in the ISO 15189 accredited medical laboratories of the hospital for further analysis. All analyses were performed in accordance with the manufacturer's recommendations. The intra- and interassay coefficients of variation for all laboratory tests were under 5%. FPG, total cholesterol, triglycerides, and high-density lipoprotein cholesterol (HDL-C) were subsequently determined with standardized enzymatic methods. Based on the new ADA guidelines of impaired FPG and dyslipidaemia, an FPG level of 5.6 to 6.9 mmol/L was considered impaired fasting glucose (IFG) [21]. A total cholesterol level of 5.18 mmol/L or greater, a triglyceride level of 1.7 mmol/L or greater, and an HDL-C level less than 1.03 mmol/L (40 mg/dL) in men or less than 1.29 mmol/L in women were considered abnormal total cholesterol, triglycerides, and HDL-C, respectively [22].

### 2.4. Construction, Evaluation, and Validation of the Nomogram

The nomogram was constructed based on the logistic regression parameter estimates in the cohort. The selection of the final model was performed using a forward selection process. Nomogram construction and validation were performed according to Iasonos's guide [23]. First, univariate models were used to regress the risk of T2D incidence on all twenty-five candidate variables, and variables with estimated regression coefficients having a statistical significance of *P* > 0.20 were removed. Then, all retained variables were included in a multivariable prediction model with forward selection and a cut-off *P* value of 0.1. In the third step, the remaining variables were included to build the final prediction model. For each model, the odds ratios (ORs) and 95% confidence intervals (95% CIs) were calculated to estimate the relative risk.

After the prediction models were developed, it was critical to evaluate their performances. Discrimination of the model was assessed using the *C*-index [24]. The calibration of the model was assessed graphically by comparing the nomogram-predicted probability to the observed probability across 10 deciles of predicted risk [25]. Calibration referred to the agreement between observed outcomes and predictions, which could be quantitatively assessed by the Hosmer-Lemeshow test and calibration plot calculated with the R package “rms.” The greater the spread between the 10 deciles, the better the nomogram could discriminate.

Sleep duration is an important risk factor for diabetes, as some studies have confirmed [26, 27]. However, few prediction models for diabetes have considered the impact of estimated sleep duration. The estimated sleep duration variable was therefore included in our study. To supplement the measured improvement in the *C*-index, two new metrics were also calculated to measure the prediction improvement with the addition of the new risk factor: integrated discrimination improvement (IDI) and net reclassification improvement (NRI) [28]. The IDI and NRI were assessed using the R package of PredictABEL [29].

Recently, decision curves [30] have been proposed to assess the population performance of risk prediction models for intervention recommendations. Decision curves are most useful when there is no consensus on the risk threshold for intervention because the curves allow one to examine risk model performance across a range of plausible risk thresholds. Therefore, decision curves are preferred over the area under the receiver operating characteristic curve. We did not account for the interaction terms between the independent variables. All continuous variables included in the model were categorized, so that the estimated contribution of these factors to diabetes risk could be expressed through nomograms.

Our literature review included 40 original articles (dated from March 2003 to December 2018) related to the development of new diabetes risk scores. Among the 22 articles identifying the risk of diabetes incidence, we selected 13 articles [31–43] for validation using our cohort. The selected articles follow the criteria below: (a) a better *C*-index; (b) the variables in the article which can be obtained in our cohort; and (c) articles from different countries and regions. The article by Wilson et al. [36] was selected for its development of the Framingham Offspring Study diabetes equations, which have been suggested to be effective in identifying those at risk for incident diabetes [44]. For the same reason, we also selected the article by Kanaya et al. [32]. We also tested the New Chinese Diabetes Risk Score [42], although it was originally developed for detecting undiagnosed diabetes.

### 2.5. Statistical Analysis

All *P* values reported are two-sided. Independent two-sample *t*-tests, chi-square tests, logistic regression analyses, and Hosmer-Lemeshow tests were performed using SAS software for Windows (version 9.2, SAS Institute Inc., Cary, NC). *C*-indexes, calibration plots, and bootstrap internal validations were performed using the Hmisc, rms, and survival ROC package in R software (version 3.3.1, R Foundation for Statistical Computing, Vienna, Austria).

## 3. Results

### 3.1. Baseline Characteristics and Follow-Up

Among 43,404 participants who were free of diabetes at baseline, 2623 (6.04%) developed diabetes over 6.83 ± 0.49 years of follow-up. People who had diabetes were more likely to have a low education level, to have a parental history of diabetes, to be overweight or obese, to have a shorter estimated daily sleep duration, or to have a higher FPG level at baseline (Table 1). The participants in the new diabetes group were more likely to have higher blood pressure, to be smokers, to be drinkers, to have a faster eating pace, to have less food for breakfast, to have less milk intake, to have salty or greasy dietary taste preferences, to have low levels of physical activity, to have greater work stress and to have higher levels of triglycerides, high-density lipoprotein, and total cholesterol ([Supplementary-material supplementary-material-1]).

### 3.2. Predictors of Incident Diabetes and Construction of the Nomograms

**Figure 2:**
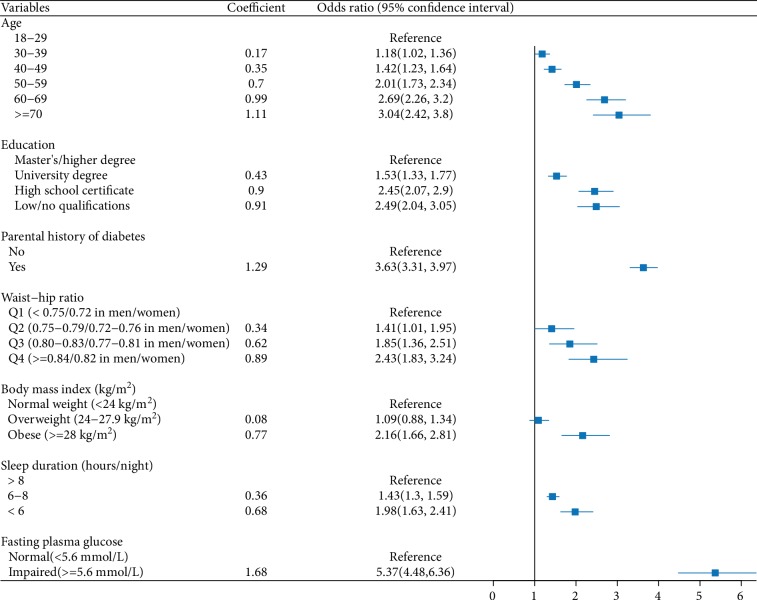
Variables involved in the multivariable logistic regression after forward selection.

Five variables were excluded (as *P* > 0.20 in the univariate analyses), including marital status, resting heart rate, dietary patterns, breakfast, and dietary preferences. Then, twenty significant variables were entered into the multivariable prediction model, and seven variables were retained after forward selection with a cut-off *P* value of 0.1 (Figure 2).

After controlling for other covariates, having a lower education level, having a family history of diabetes, having a higher waist-hip ratio, being overweight or obese, having shorter estimated daily sleep durations, and having impaired FPG were significantly associated with a higher risk of incident diabetes. Compared with those who slept more than 8 h per day, individuals who had an estimated sleep duration of 6–8 or <6 h had an increased risk of diabetes incidence (respectively, odds ratio (OR) = 1.43, 95%CI = 1.30-1.59; OR = 1.98, 95%CI = 1.63-2.41).

**Figure 3:**
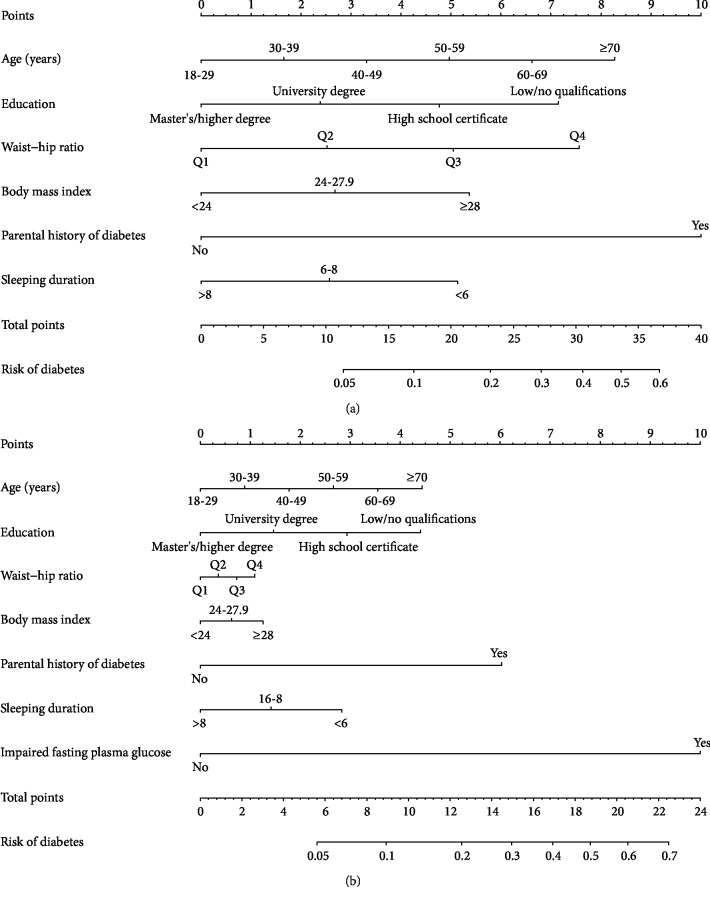
The nomograms of incident diabetes at *t* = 7 years from the Beijing Health Management Cohort study population (a) in the concise nomogram and (b) in the comprehensive nomogram. Q1 (quantile 1): <0.75 in men or <0.72 in women; Q2 (quantile 2): 0.75-0.79 in men or 0.72-0.76 in women; Q3 (quantile 3): 0.80-0.83 in men or 0.77-0.81 in women; Q4 (quantile 4): ≥0.84 in men or ≥0.82 in women.

Finally, two nomograms were developed to predict the 7-year risk of diabetes (Figure 3). The comprehensive nomogram included age, education, parental history of diabetes, waist-hip ratio, BMI, sleep duration, and FPG. FPG was excluded to produce the concise nomogram. We determined nomogram score cut-off values with which participants were evenly stratified into five risk groups: very low risk, low risk, moderate risk, high risk, and very high risk (Table 2). This stratification effectively discriminated the diabetes incidence for the five proposed risk groups (*P* for trend < 0.001).

### 3.3. Calibration, Discrimination, and Internal Validation of the Nomogram

**Figure 4:**
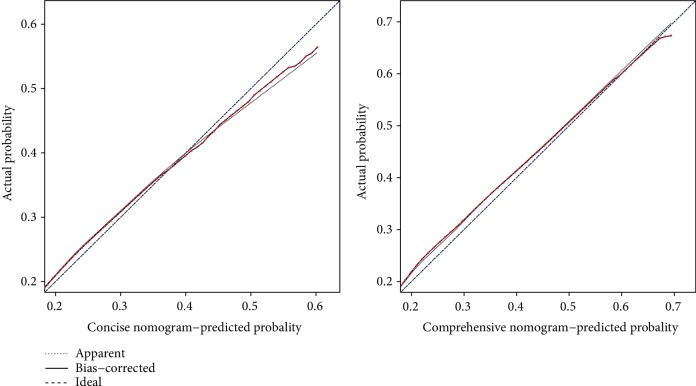
Calibration plots by deciles for nomograms: 7-year incidence of diabetes in the concise nomogram and the comprehensive nomogram, respectively. (The nomogram-predicted probabilities of diabetes incidence are plotted on the *x*-axis; actual probabilities of diabetes incidence are plotted on the *y*-axis. Dashed lines along the 45-degree line through the origin point represent perfect calibration models, in which the predicted probabilities are identical to the actual probabilities.)

The total scores for the concise nomogram varied from 0 to 43, and those for the comprehensive nomogram varied from 0 to 29. The calibration plot for the probability of incident diabetes showed good calibration (Hosmer-Lemeshow test, chi-square = 15.506, *P* = 0.050; chi-square = 12.626, *P* = 0.125), and the actual diabetes risk in the cohort was similar to the predicted risk (Figure 4). The *C*-index demonstrated that both the concise and the comprehensive nomograms had medium-high predictive capacity (Table 3). After internal validation by bootstrapping, the optimism-corrected *C*-index of the concise and comprehensive nomograms for the test set was 0.73 (95% CI: 0.71-0.75) and 0.74 (95% CI: 0.71 0.76), respectively, suggesting well-validated models.

### 3.4. Additional Improvement from Estimated Sleep Duration

**Figure 5:**
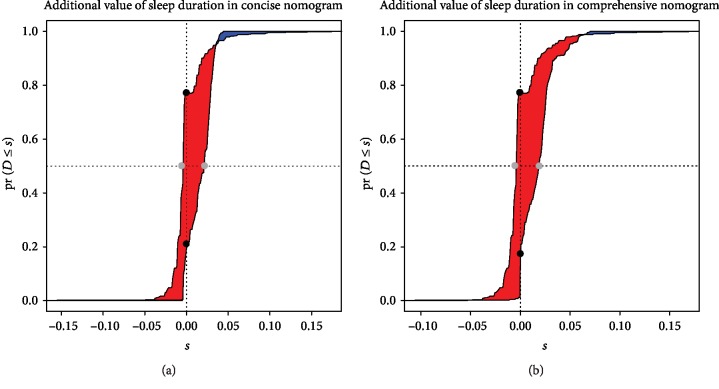
The additional value of sleep duration, as assessed by the paired difference of nomograms (a) in the concise nomogram and (b) in the comprehensive nomogram.

The additional value of estimated sleep duration was assessed by the paired difference of risk scores. The empirical distribution function of the change in estimated risk scores for subjects who had diabetes (thick solid line) and those who were free of diabetes (thin solid line) was assessed (Figure 5). The difference between the areas under the two curves is the IDI, and the distances between the two black dots and between the two grey dots represent the continuous NRI and median improvement, respectively. For the concise score, the IDI and NRI values of estimated sleep duration were 0.016 (95% CI: 0.011-0.020, *P* < 0.001) and 0.597 (95% CI: 0.520-0.659, *P* < 0.001), respectively; the median increment of estimated sleep duration was 0.022 (95% CI: 0.016-0.027; *P* < 0.001). For the comprehensive score, the IDI and NRI values of estimated sleep duration were 0.017 (95% CI: 0.012-0.022, *P* < 0.001) and 0.619 (95% CI: 0.518-0.695, *P* < 0.001), respectively; the median increment of estimated sleep duration was 0.020 (95% CI: 0.014-0.025, *P* < 0.001).

### 3.5. Clinical Utility of the Nomograms

**Figure 6:**
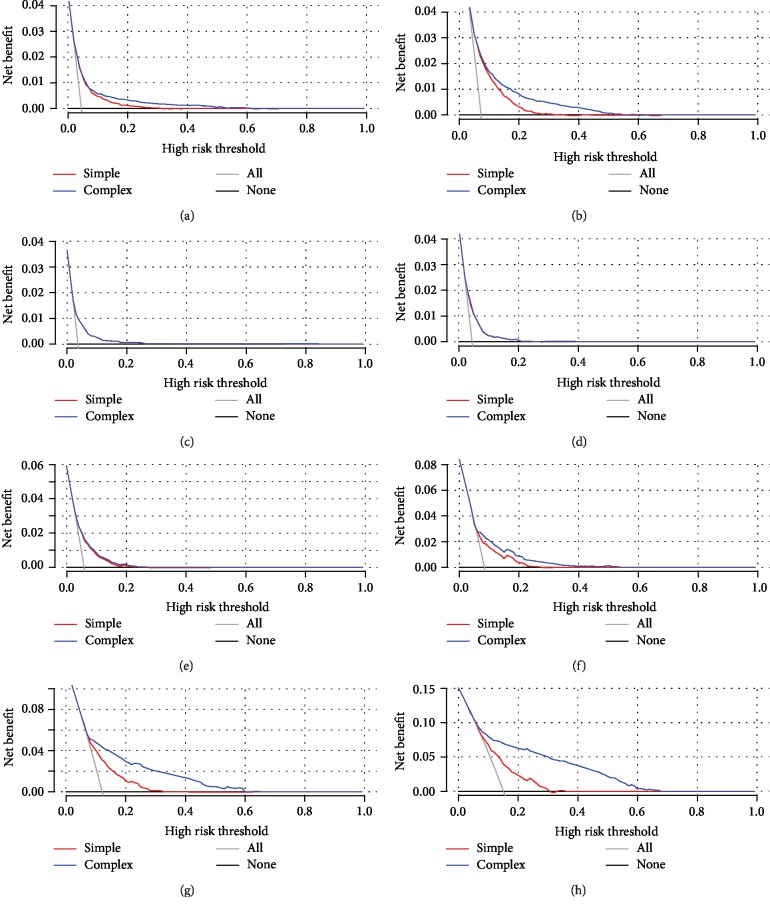
The performance of concise nomogram and comprehensive nomogram scores in the following subpopulations: (a) male, (b) female, (c) 18-29 years old, (d) 30-39 years old, (e) 40-49 years old, (f) 50-59 years old, (g) 60-69 years old, and (h) ≥70 years old.

The population decision curves showed that the two risk scores were similar in the population. The subpopulation decision curves showed that for women or individuals older than 50 years, the subpopulation net benefit (NB) was significantly higher for the comprehensive score (blue line) (Figure 6). For men or individuals younger than 50 years, the two scores have a similar low NB.

### 3.6. Validation of Other Existing Risk Scores

Table 4 summarizes the performance of 13 existing diabetes scores, including eight scores containing laboratory variables [32, 33, 36, 38–41, 43] and five scores that do not [31, 34, 35, 37, 42]. When applied to our cohort, none of the 5 scores that do not contain laboratory variables outperformed our comprehensive score (Table 4). In terms of discrimination and calibration, our comprehensive score containing laboratory variables also performed better than the other 8 scores that also contained laboratory variables. Among the 13 risk scores, the diabetes risk score developed by Wang et al. [43] performed the best (*C*-index of 0.75, *P* = 0.214 for calibration).

## 4. Discussion

The current study links the development of T2D and sleep duration in urban Chinese adults after adjusting for other known major T2D risk factors. Short sleep duration was significantly associated with an increased risk of developing T2D. We have constructed two nomograms to predict individualized T2D risk that consider the influence of sleep duration and provide a practical guide to identify adults at a high risk for T2D. The comprehensive nomogram performed better than the concise nomogram. Women or individuals older than 50 years had greater benefit from the comprehensive nomogram for predicting T2D risk.

This study extends and expands on the existing diabetes scores by adding a novel factor, namely, estimated sleep duration. To the best of our knowledge, this is the first study to include estimated sleep duration in seven-year risk nomograms for T2D. The estimated sleep duration was assigned 3 points in the comprehensive nomogram. The association between a short self-reported sleep duration and T2D could be confounded by BMI, or sleep restriction may mediate its effects on T2D through weight gain [45]. Our study showed that shorter sleep duration was an independent risk factor for T2D after adjustments were made for BMI, which is consistent with other research findings [26, 27]. Several potential biological mechanisms may contribute to the relationship between short sleep duration and T2D. First, laboratory studies have corroborated the decreases in glucose tolerance and insulin sensitivity after sleep restriction [46]. Both inadequate pancreatic insulin secretion and increased circulating levels of glucose due to sleep deprivation could lead to the development of insulin resistance and type 2 diabetes [47, 48]. Changes in the activity of neuroendocrine systems seem to be major mediators of the detrimental metabolic effects of sleep restriction [10]. Second, short sleep duration was associated with increases in inflammation markers [49], such as interleukin-6 and C-reactive protein, which indicate low-level systemic inflammation and play a role in T2D development [50]. It is possible that sleep disruption is related to T2D via a mechanism of low-grade systemic inflammation. Finally, short sleep is associated with increases in ghrelin and decreases in leptin, leading to a longer eating time, thereby increasing the risk of weight gain and subsequent health risks [48, 51].

Other variables included in the comprehensive nomogram are age, education level, WHR, BMI, parental history of diabetes, and FPG. The FPG variable was the strongest predictor of incident T2D (a contribution of 10 points). This finding is roughly consistent with those of previous studies [52]. The risk of T2D incidence increased with higher FPG levels. A parental history of diabetes was the second-strongest predictor after FPG (a contribution of 6 points), and genetic and environmental pathways may be able to account for this association. Age and education level were the third-strongest predictors after parental history of T2D (a contribution of 4 points each). The inclusion of education level in the diabetes score is also common. A previous study demonstrated an inverse association between educational level, risk of diabetes, and inequalities in the risk of diabetes in Western European countries. BMI had a contribution of 1 point and has also been observed in most of the published T2D scores [53]. WHR is also an important predictor of incident diabetes in our scores.

The concise nomogram is noninvasive and can be administered by the individuals themselves. The comprehensive nomogram is more effective but requires simple blood tests. In terms of discrimination and calibration, both nomograms performed better compared with 13 risk scores derived from other populations. The subgroup analysis showed that women or people older than 50 years had the largest net benefit in the decision curves. Furthermore, the clinical variables that we have chosen to incorporate into the nomograms can be easily documented by any physician and the participants, enhancing its practical utility.

Previously, the Finnish Diabetes Risk Score (FINDRISK) [31], the German Diabetes risk score [35], and the Framingham DM risk score [36] were considered the most widely useful scores in the clinical guidelines. The *C*-indexes of these diabetes risk scores for adults ranged from 0.62 to 0.87 in their original population [31, 42] and ranged from 0.68 to 0.75 in the current study population. Both our concise and comprehensive nomograms performed with a moderately high *C*-index value (0.73 and 0.74, respectively) in the test set. All predictors included in our nomograms are readily available clinical variables. Based on the nomograms, we further stratify patients into five distinct risk groups. Trend analysis implied that there was a statistically significant linear correlation between the scores based on nomograms and actual diabetes risk.

Our research has some advantages. First, our sample size was relatively large. Second, comprehensive novel factors, such as estimated sleep duration, eating pace, and taste preferences, were taken into account in our research. Third, nomograms and decision curves were used to visualize the risk score to improve its clinical utility. Fourth, multiple novel methods were used to evaluate the performance of the nomograms, including calibration plots, decision curves, IDI, and NRI. The calibration plots showed optimal agreements between the prediction and actual observation of T2D cases, which guarantees the reliability of the established nomograms. Decision curves helped to identify the most suitable population for application. According to the IDI and NRI values, the prediction model that included estimated sleep duration was superior to the model without this variable.

There were also some limitations to our research. First, we were not able to include oral glucose tolerance test data, additional sleep dimensions or characteristics (such as sleep quality, apnoea, and insomnia), and longitudinal changes in sleep duration, which could affect T2D outcomes and impact sleep. Second, our research relied on self-reported sleep duration, whereas objective methods (such as actigraphy and polysomnography) might provide more accurate measures. However, objective measures of sleep duration are too expensive to be feasible in large prospective cohort studies. In addition, several validation studies observed a high correlation (*r* = 0.79-0.95) between self-reported and actigraphy-measured sleep duration [54, 55]. Third, this study was conducted using a sample of the Beijing population (mean age: 36.79 ± 13.29 years; age range: 18–80 years; 55.31% males; 7-year incidence of T2D 6.04%), which had a higher socioeconomic status (82.2% had a bachelor degree or higher) and higher prevalence of major risk factors (57.4% smokers) compared with the general population. Thus, our prediction models might have limited generalizability to other populations. Another limitation is that the lack of external validation may limit the extrapolation of the scores. Although all results consistently show the satisfactory performance of the established nomograms, additional external validation in prospective datasets in future studies is warranted. Fourth, a follow-up bias may have easily occurred in the long-term follow-up. However, the sensitivity analysis showed that there were no significant differences in the distributions of baseline characteristics between those individuals who lost to follow-up and those who were followed. Finally, as the questionnaire was not validated and was self-reported, a measurement bias is inevitable.

## 5. Conclusions

Short sleep duration was associated with increased T2D risk in adults. Increased estimated sleep duration could improve the performance of T2D risk nomograms. Our nomograms are more suitable for predicting T2D risk for women or individuals ≥ 50 years old. Convincingly, tailored interventions and preventive strategies could be readily developed to decrease the risk of diabetes.

## Figures and Tables

**Table 1:** Baseline characteristics between participants of incident diabetes and nondiabetes of selected factors in the model (*N* = 43,404).

Characteristic	No diabetes (*n* = 40781)	New diabetes (*n* = 2623)	*t*/*χ*^2^	*P*
Mean age	36.4 ± 13.1	42.7 ± 15.1	-23.58	<0.001
Sex (% men)	22254 (54.6)	1753 (66.8)	149.92	<0.001
Marital status				
Never married/divorced/widowed	5542 (13.6)	327 (12.5)	2.66	0.103
Married	35239 (86.4)	2296 (87.5)		
Education				
Master's/higher degree	6447 (15.8)	226 (8.6)	236.44	<0.001
University degree	27340 (67.0)	1679 (64.0)		
High school certificate	4872 (11.9)	476 (18.1)		
Low/no qualifications	2122 (5.2)	242 (9.2)		
Parental history of diabetes				
Yes	7064 (17.3)	955 (36.4)	596.10	<0.001
No	33717 (82.7)	1668 (63.6)		
Mean resting heart rate (beats per minute)	79.9 ± 9.2	80.8 ± 9.7	-2.05	0.040
Sleep duration (hours/night)				
<6	1101 (2.7)	128 (4.9)	257.51	<0.001
6-8	11923 (29.2)	1102 (42.0)		
>8	27757 (68.1)	1393 (53.1)		
Eating pace				
Slow	4072 (10.0)	171 (6.5)	54.59	<0.001
Medium	16614 (40.7)	995 (37.9)		
Fast	20095 (49.3)	1457 (55.5)		
Breakfast frequency				
Never	4946 (12.1)	347 (13.2)	24.48	<0.001
≤2 times/week	4095 (10.0)	208 (7.9)		
3-6 times/week	5385 (13.2)	294 (11.2)		
≥7 times/week	26355 (64.6)	1774 (67.6)		
Taste preferences				
Light	20945 (51.4)	1220 (46.5)	64.31	<0.001
Salty	9335 (22.9)	762 (29.1)		
Greasy	4594 (11.3)	324 (12.4)		
Sweet	4283 (10.5)	225 (8.6)		
Others	1624 (4.0)	92 (3.5)		
Mean waist-hip-ratio	0.8 ± 0.1	0.9 ± 0.1	-19.85	<0.001
Body mass index				
Normal weight (<24 kg/m^2^)	38468 (94.4)	2287 (87.2)	308.32	<0.001
Overweight (24-27.9 kg/m^2^)	1726 (4.2)	201 (7.7)		
Obese (≥28 kg/m^2^)	536 (1.3)	134 (5.1)		
Fasting plasma glucose (SD)	4.7 ± 0.5	5.7 ± 0.7	-37.33	<0.001
Fasting plasma glucose				
Impaired (≥5.6 mmol/L)	313 (0.8)	271 (10.3)	1698.33	<0.001
Normal (<5.6 mmol/L)	40468 (99.2)	2352 (89.7)		

Data are % or the means ± Standard Deviation.

**Table 2:** Nomograms scores to stratify the 7-year risk of type 2 diabetes.

Subgroups	Concise nomogram		Comprehensive nomogram	
	Score	Estimated incidence (%)	Score	Estimated incidence (%)
Very low risk	<7	2.33	<2	2.32
Low risk	7-8	3.22	2-3	3.15
Moderate risk	8-12	4.25	3-5	4.13
High risk	12-17	7.40	5-8	7.90
Very high risk	>17	15.11	>8	14.74
*P* for trend	<0.001		<0.001	

**Table 3:** The *C*-index (95% CI) of the nomograms after bootstrapping validation.

	Concise nomogram		Comprehensive nomogram	
	Training set	Test set	Training set	Test set
Male	0.73 (0.72-0.75)	0.73 (0.72-0.74)	0.73 (0.72-0.74)	0.73 (0.71-0.75)
Female	0.73 (0.72-0.74)	0.73 (0.72-0.75)	0.74 (0.72-0.76)	0.74 (0.71-0.77)
Age < 60	0.69 (0.62-0.72)	0.69 (0.61-0.77)	0.74 (0.73-0.75)	0.72 (0.70-0.75)
Age ≥ 60	0.73 (0.72-0.74)	0.72 (0.70-0.75)	0.75 (0.70-0.79)	0.80 (0.75-0.83)
Total	0.73 (0.72-0.75)	0.73 (0.71-0.75)	0.74 (0.73-0.75)	0.74 (0.71-0.76)

**Table 4:** The performance of existing scores in predicting incident diabetes in the Beijing Health Management Cohort study.

Year	Leading author	Population	Predictors	Validation in the Beijing Health Management Cohort study	
				*C*-index (95% CI)	*P* value of Hosmer-Lemeshow test
2003	Lindstrom	Finnish	^‡^Age, BMI, waist circumference, hypertension, ^§^history of high blood glucose	0.69 (0.68, 0.70)	0.560
2005	Kanaya	American	Age, sex, TG, FPG	0.68 (0.67, 0.69)	0.173
2005	Schmidt	American	^||^Age, race, parental history of diabetes, FPG, SBP, waist circumference, height, HDL-C, TG	0.74 (0.73, 0.75)	0.269
2006	Aekplakorn	Thai	^¶^Age, sex, BMI, waist circumference, hypertension, history of diabetes in parent or sibling	0.74 (0.73, 0.75)	0.404
2007	Schulze	German	Age, waist circumference, height, moderate alcohol, smoking, (red meat, whole-grain bread, coffee, ^#^physical activity)	0.71 (0.70, 0.72)	0.002
2007	Wilson	American	^∗^BMI, parental history of diabetes, hypertension, HDL-C, TG, FPG	0.71 (0.70, 0.72)	0.197
2008	Balkau	French	^†^Men: waist circumference, smoking, hypertension Women: waist circumference, diabetes in the family, hypertension	Men: 0.71 (0.70, 0.72) Women: 0.69 (0.68, 0.70)	Men: 0.672 Women: 0.965
2009	Chien	Chinese	Age, BMI, WBC, TG, HDL-C, FPG	0.69 (0.67, 0.70)	0.005
2009	Gao	Indian	^‡‡^Age, sex, BMI, waist circumference, FPG, TG	0.69 (0.67, 0.70)	0.115
2009	Kahn	American	^##^Age, parental history of diabetes, hypertension, race, drinking, waist circumference, height, resting pulse, FPG, TG, HDL-C, UA	0.74 (0.73, 0.75)	0.424
2010	Chen	Australian	Age, sex, BMI, race, waist circumference, parental history of diabetes, history of high blood glucose, hypertension, smoking, physical inactivity	0.75 (0.74, 0.76)	0.029
2013	Zhou	Chinese	Age, sex, BMI, waist circumference, SBP, family history of diabetes	0.75 (0.74, 0.76)	0.029
2016	Wang	Chinese	Age, sex, BMI, family history of diabetes, education, hypertension, resting heart rate, FPG, TG	0.75 (0.74, 0.76)	0.214

The variables in parentheses were removed from the original model in validation because they could not be provided in sufficient detail in the Beijing Health Management Cohort study. BMI: body mass index; FPG: fasting plasma glucose; SBP: systolic blood pressure; HDL-C: high-density lipoprotein cholesterol; TG: triglyceride; WBC: white blood cell; UA: uric acid.

## Data Availability

The data that support the findings of this study are available from the Beijing Health Management Cohort (BHMC) study, but restrictions apply to the availability of these data, which were used under license for the current study and so are not publicly available. Data are however available from the authors upon reasonable request and with permission of the Capital Medical University.

## Supplementary Materials

Supplementary Materials Table S1 shows the comparison of baseline characteristics (not included in the model) between no diabetes and new diabetes groups. The new diabetes group was more likely to have higher blood pressure, to be smokers, to be drinkers, to have a faster eating pace, to have less food for breakfast, to have less milk intake, to have salty or greasy dietary taste preferences, to have low levels of physical activity, to have greater work stress, and to have higher levels of triglycerides, high-density lipoprotein, or total cholesterol.
