# Supplementary material for: A Novel Risk Score for Type 2 Diabetes Containing Sleep Duration: A 7-Year Prospective Cohort Study among Chinese Participants
Source: J Diabetes Res. 2020 Jan 4;2020:2969105. doi: 10.1155/2020/2969105 (PMC6964717; doi:10.1155/2020/2969105)
Supplement: Supplementary Materials — Table S1 shows the comparison of baseline characteristics (not included in the model) between no diabetes and new diabetes groups. The new diabetes group was more likely to have higher blood pressure, to be smokers, to be drinkers, to have a faster eating pace, to have less food for breakfast, to have less milk intake, to have salty or greasy dietary taste preferences, to have low levels of physical activity, to have greater work stress, and to have higher levels of triglycerides, high-density lipoprotein, or total cholesterol. [file 2969105.f1.docx]

## Supplementary Materials

Table S1 shows the comparison of baseline characteristics (not included in the model) between no diabetes and new diabetes groups. The new diabetes group were more likely to have higher blood pressure, to be smokers, to be drinkers, to have a faster eating pace, to have less food for breakfast, to have less milk intake, to have salty or greasy dietary taste preferences, to have low levels of physical activity, to have greater work stress, and to have higher levels of triglycerides, high density lipoprotein, or total cholesterol.

**Table S1**. Baseline characteristics (not included in the model) between groups.

| Characteristic | No diabetes  (n=40781) | New diabetes  (n=2623) | t/$\chi^{2}$ | *P* |
| --- | --- | --- | --- | --- |
| Blood pressure (mmHg) |  |  |  |  |
| Systolic blood pressure <140 and diastolic blood pressure <90 | 39837 (97.7) | 2439 (93.0) | 215.08 | <0.001 |
| Systolic blood pressure≥140 and/or diastolic blood pressure≥90 | 944 (2.3) | 184 (7.0) |  |  |
| Passive smoking status |  |  |  |  |
| No | 28831 (70.7) | 2031 (77.4) | 54.38 | <0.001 |
| Yes | 11950 (29.3) | 592 (22.6) |  |  |
| Active smoking status |  |  |  |  |
| Never smoker | 17747 (43.5) | 752 (28.7) | 293.79 | <0.001 |
| 1-5 cigarettes/day | 17195 (42.2) | 1285 (49.0) |  |  |
| 6-20 cigarettes/day | 1259 (3.1) | 176 (6.7) |  |  |
| >21 cigarettes/day | 4580 (11.2) | 410 (15.6) |  |  |
| Drinking frequencies |  |  |  |  |
| Never | 13375 (32.8) | 584 (22.3) | 266.99 | <0.001 |
| <1.5 kg/month | 21586 (52.9) | 1411 (53.8) |  |  |
| 1.5-3 kg/month | 4158 (10.2) | 399 (15.2) |  |  |
| >3 kg/month | 1662 (4.1) | 229 (8.7) |  |  |
| Milk intake |  |  |  |  |
| >7 times/week | 11197 (27.5) | 1022 (39.0) | 176.21 | <0.001 |
| 1-2 times/week | 19280 (47.3) | 1002 (38.2) |  |  |
| 3-5 times/week | 6952 (17.0) | 363 (13.8) |  |  |
| 6-7 times/week | 3352 (8.2) | 236 (9.0) |  |  |
| Grains intake |  |  |  |  |
| More coarse grains | 1949 (4.8) | 167 (6.4) | 16.62 | <0.001 |
| Balanced | 10947 (26.8) | 734 (28.0) |  |  |
| More refined grains | 27885 (68.4) | 1722 (65.7) |  |  |
| Dietary patterns |  |  |  |  |
| Balanced | 28720(77.6) | 1832 (76.0) | 4.02 | 0.134 |
| More meat | 7813 (21.1) | 541 (22.4) |  |  |
| More vegetarian | 465 (1.3) | 37 (1.5) |  |  |
| Physical activity intensity |  |  |  |  |
| Large | 5158 (12.6) | 232 (8.8) | 52.80 | <0.001 |
| Medium | 11867 (29.1) | 774 (29.5) |  |  |
| Mild | 10911 (26.8) | 831 (31.7) |  |  |
| None | 12845 (31.5) | 786 (30.0) |  |  |
| Work stress |  |  |  |  |
| Small | 11029 (27.0) | 519 (19.8) | 209.28 | <0.001 |
| Medium | 15384 (37.7) | 930 (35.5) |  |  |
| Large | 13334 (32.7) | 1006 (38.4) |  |  |
| Retired | 1034 (2.5) | 168 (6.4) |  |  |
| Triglycerides (SD, mmol/L) | 2.4 ± 1.0 | 2.0 ± 1.1 | 8.28 | <0.001 |
| Triglycerides |  |  |  |  |
| <1.7 mmol/L | 36663 (89.9) | 2314 (88.2) | 7.62 | 0.006 |
| ≥1.7 mmol/L | 4118 (10.1) | 309 (11.8) |  |  |
| High-density lipoprotein cholesterol (mmol/L) | 1.2 ± 0.2 | 1.2 ± 0.2 | -0.02 | 0.988 |
| High-density lipoprotein cholesterol (mmol/L, %) |  |  |  |  |
| ≥1.03/1.29 in men/women | 23346 (57.2) | 1836 (70.0) | 164.46 | <0.001 |
| <1.03/1.29 in men/women | 17435 (42.8) | 787 (30.0) |  |  |
| Total cholesterol (SD, mmol/L) | 4.5 (0.9) | 4.9 (0.9) | -7.71 | <0.001 |
| Total cholesterol |  |  |  |  |
| <5.18 mmol/L | 40238 (98.7) | 2538 (96.8) | 62.99 | <0.001 |
| ≥5.18 mmol/L | 543 (1.3) | 85 (3.2) |  |  |
